# Privacy-preserving distributed learning of radiomics to predict overall survival and HPV status in head and neck cancer

**DOI:** 10.1038/s41598-020-61297-4

**Published:** 2020-03-11

**Authors:** Marta Bogowicz, Arthur Jochems, Timo M. Deist, Stephanie Tanadini-Lang, Shao Hui Huang, Biu Chan, John N. Waldron, Scott Bratman, Brian O’Sullivan, Oliver Riesterer, Gabriela Studer, Jan Unkelbach, Samir Barakat, Ruud H. Brakenhoff, Irene Nauta, Silvia E. Gazzani, Giuseppina Calareso, Kathrin Scheckenbach, Frank Hoebers, Frederik W. R. Wesseling, Simon Keek, Sebastian Sanduleanu, Ralph T. H. Leijenaar, Marije R. Vergeer, C. René Leemans, Chris H. J. Terhaard, Michiel W. M. van den Brekel, Olga Hamming-Vrieze, Martijn A. van der Heijden, Hesham M. Elhalawani, Clifton D. Fuller, Matthias Guckenberger, Philippe Lambin

**Affiliations:** 1 University Hospital Zurich and University of Zurich, Department of Radiation Oncology, Zurich, Switzerland; 2 GROW–School for Oncology and Developmental Biology-Maastricht University Medical Centre-, Department of Precision Medicine, The D Lab: Decision Support for Precision Medicine-, Maastricht, The Netherlands; 3 Princess Margaret Cancer Center- University of Toronto, Department of Radiation Oncology, Toronto, Ontario Canada; 4 Kantonsspital Aarau, Center for Radiation Oncology- KSA-KSB-, Aarau, Switzerland; 5 Cantonal Hospital Lucerne, Radiation Oncology, Lucerne, Switzerland; 6 Amsterdam UMC, Vrije Universiteit Amsterdam, Department of Otolaryngology/Head and Neck Surgery, Amsterdam, The Netherlands; 7 Parma University Hospital, Radiology Department, Parma, Italy; 8 IRCCS Fondazione Istituto Nazionale dei Tumori, Radiology Department, Milan, Italy; 9 University Hospital Duesseldorf, Heinrich-Heine-University, Department of Otorhinolaryngology & Head/Neck, Surgery, Duesseldorf, Germany; 10 Department of Radiation Oncology (MAASTRO), GROW-School for Oncology and Developmental Biology-Maastricht University Medical Centre, Department of Radiation Oncology, Maastricht, The Netherlands; 11 Amsterdam UMC, Vrije Universiteit Amsterdam, Department of Radiation Oncology, Amsterdam, The Netherlands; 12 University Medical Center Utrecht, Department of Radiotherapy, Utrecht, The Netherlands; 13 The Netherlands Cancer Institute, Department of Head and Neck Oncology and Surgery, Amsterdam, The Netherlands; 14 The Netherlands Cancer Institute, Department of Radiation Oncology, Amsterdam, The Netherlands; 15 Department of Radiation Oncology, The University of Texas MD Anderson Cancer Center, Houston, TX USA

**Keywords:** Prognostic markers, Head and neck cancer, Computational science

## Abstract

A major challenge in radiomics is assembling data from multiple centers. Sharing data between hospitals is restricted by legal and ethical regulations. Distributed learning is a technique, enabling training models on multicenter data without data leaving the hospitals (“privacy-preserving” distributed learning). This study tested feasibility of distributed learning of radiomics data for prediction of two year overall survival and HPV status in head and neck cancer (HNC) patients. Pretreatment CT images were collected from 1174 HNC patients in 6 different cohorts. 981 radiomic features were extracted using Z-Rad software implementation. Hierarchical clustering was performed to preselect features. Classification was done using logistic regression. In the validation dataset, the receiver operating characteristics (ROC) were compared between the models trained in the centralized and distributed manner. No difference in ROC was observed with respect to feature selection. The logistic regression coefficients were identical between the methods (absolute difference <10^−7^). In comparison of the full workflow (feature selection and classification), no significant difference in ROC was found between centralized and distributed models for both studied endpoints (DeLong p > 0.05). In conclusion, both feature selection and classification are feasible in a distributed manner using radiomics data, which opens new possibility for training more reliable radiomics models.

## Introduction

In recent years radiomics has shown to be a promising tool in disease classification and prognostic modeling^1–4^. One of the major challenges in radiomics is assembling a large cohort, which is essential for reliable model training. Training models on small cohorts without validation can result in model overfitting and lack of generalization^5,6^. It is difficult to collect a sufficiently large amount of data in a single institution setting. Single institution data may also not represent variations in patient populations across the world. Moreover, single institution data may not be a good representation of global variations in image acquisition protocols, which further influence quantitative image analysis^7^. On the other hand, sharing data between hospitals is restricted by legal and ethical regulations^8,9^. Patients signing an informed consent should have two options: participating in the study in a full extent or participating in a study without external data sharing^10^. Additionally, central collection of imaging data requires large storage infrastructure.

Distributed learning in radiotherapy, introduced in 2013 and pioneered in the euroCAT network, is a promising technique to address these challenges^11^. This methodology allows for training a model on data, which do not leave a local repository, for example a hospital. Instead, the model parameters are sent between members of the network and the central server. These models parameters are aggregate values and cannot be reversed or linked back to individual data points. Hence, this approach has also been referred to as “privacy-preserving” distributed learning^12^. Results from different members are compared in the central server and the updated results are sent back to the members. This procedure is continued until an agreement is reached. The feasibility of distributed learning for training prognostic models in healthcare was already shown for prediction of both normal tissue complications and overall survival following radiotherapy^12–14^. The prognostic power of the models trained in the distributed fashion was equally good as the models trained in the centralized manner.

In previously published works, the sole process of model fitting and data privacy issues were investigated. However, training a radiomics-based model requires two additional steps: feature normalization and feature selection. Feature normalization can be done with the assumption of selecting random samples (single hospital data) from a normal distribution (overall population). Radiomic features are known to exhibit a high degree of correlation and thus dimensionality reduction is a crucial step of the radiomics workflow. Distributed feature selection algorithms for horizontal data partitioning have been investigated^15,16^. In horizontal partitioning, the database is split based on rows, where each smaller database has the same structure. This type of feature selection was not tested on radiomics data. Therefore, this work aims at developing and testing a distributed learning workflow for model training on radiomics data. We hypothesize that distributed algorithms can be used to efficiently train robust radiomics models, achieving quality comparable with models trained in a centralized manner. We have used data from six different head and neck cancer (HNC) cohorts (more than 1000 patients) to compare results from centralized and distributed workflows. The workflows were evaluated on two, clinically-relevant binary endpoints, tumor human papillomavirus (HPV) status and 2 year overall survival.

## Material and methods

### Analyzed cohorts

This retrospective analysis was based on 6 cohorts of patients, with a total enrollment of 1174 patients. The analysis was approved by local ethical commissions and was conducted according to their guidelines, for some cohorts the need for informed consent was waived (see details in the Supplement). The survival data were available for 1064 patients from 5 different cohorts. Similarly, HPV status was determined in biopsy analysis in 834 patients from 5 cohorts. Details on the studied cohorts can be found in Table 1 and imaging protocols are described in the Table 1S. The HPV status was confirmed by immunohistochemical p16 staining in biopsy specimens. All patients were treated with definitive chemoradiotherapy, except the VUmc and PMH cohort, where definitive radiotherapy alone was allowed. The patients underwent contrast-enhanced CT imaging for the purpose of treatment planning, according to the local protocols.

**Table 1:** Characteristic of studied cohorts.

	Center	BD2Decide	Design	MD Anderson	PMH	VUmc	USZ
	number of patients	206	141	110	441	100	176
2 years OS	dead	55 64%	36 66%	0 0%	96 72%	31 55%	40 71%
	alive	151 36%	105 34%	0 0%	345 28%	69 45%	136 29%
	unknown	0 0%	0 0%	110 100%	0 0%	0 0%	0 0%
HPV	positive	33 16%	0 0%	98 89%	274 62%	23 23%	58 33%
	negative	61 30%	141 100%	12 11%	116 26%	77 77%	82 47%
	unknown	112 54%	0 0%	0 0%	51 12%	0 0%	36 20%
Head and neck tumor site	oropharynx	128 62%	63 45%	110 100%	441 100%	100 100%	113 64%
	hypopharynx	13 6%	47 33%	0 0%	0 0%	0 0%	37 21%
	larynx	20 10%	31 22%	0 0%	0 0%	0 0%	16 9%
	oral cavity	45 22%	0 0%	0 0%	0 0%	0 0%	10 6%

### Radiomics analysis

Radiomic features were extracted from the primary tumor region. The treatment defined gross tumor volume (GTV) was visually assessed for the presence of artifacts and slices with artifacts were manually removed from the contour. Images were resampled to 3.3 mm cubic voxels using linear interpolation. The Hounsfield unit range was set to (−20, 180) to limit the analysis to soft tissue. In total, 981 features were extracted with the Z-Rad radiomics software implementation^17^:

- shape (n = 18).
- intensity distribution (n = 17).
- texture (n = 90): the Gray Level Co-occurrence Matrix (n = 26), the Neighborhood Gray Tone Difference Matrix (n = 4), the Gray Level Run Length Matrix (n = 14), the Gray Level Size Zone Matrix (n = 14), the Gray Level Distance Zone Matrix (n = 16) and the Neighboring Gray Level Dependence Matrix (n = 16).
- wavelet transform (n = 856).

### Distributed learning platform

The Oncoradiomics distributed learning solution DistriM was used. This software consists of a master script and a site script. The site script is executed at each medical institution, where the data is located, and waits for a learning call from the master script. The master script is run by the researcher and initiates the distributed learning procedure. This script also mediates the transmission of the model coefficients to and from the sites. When model learning is complete, the master script outputs the model coefficients of the learned model. In this experimental setting, all data was centralized and artificially distributed across laptops on a per-center basis. The site script was executed on each laptop. The laptops were located at Maastricht University.

### Feature selection

First, data quality check was performed. Missing values were assessed and features with more than 20% missing values were excluded. Similarly, to avoid outliers, features with skewed distribution (skewness > 5) were excluded. The exclusion criteria were evaluated in the entire dataset for the centralized learning and per cohort for distributed learning. In the distributed learning, the union of features excluded per cohort was considered as the excluded subset.

**Figure 1:**
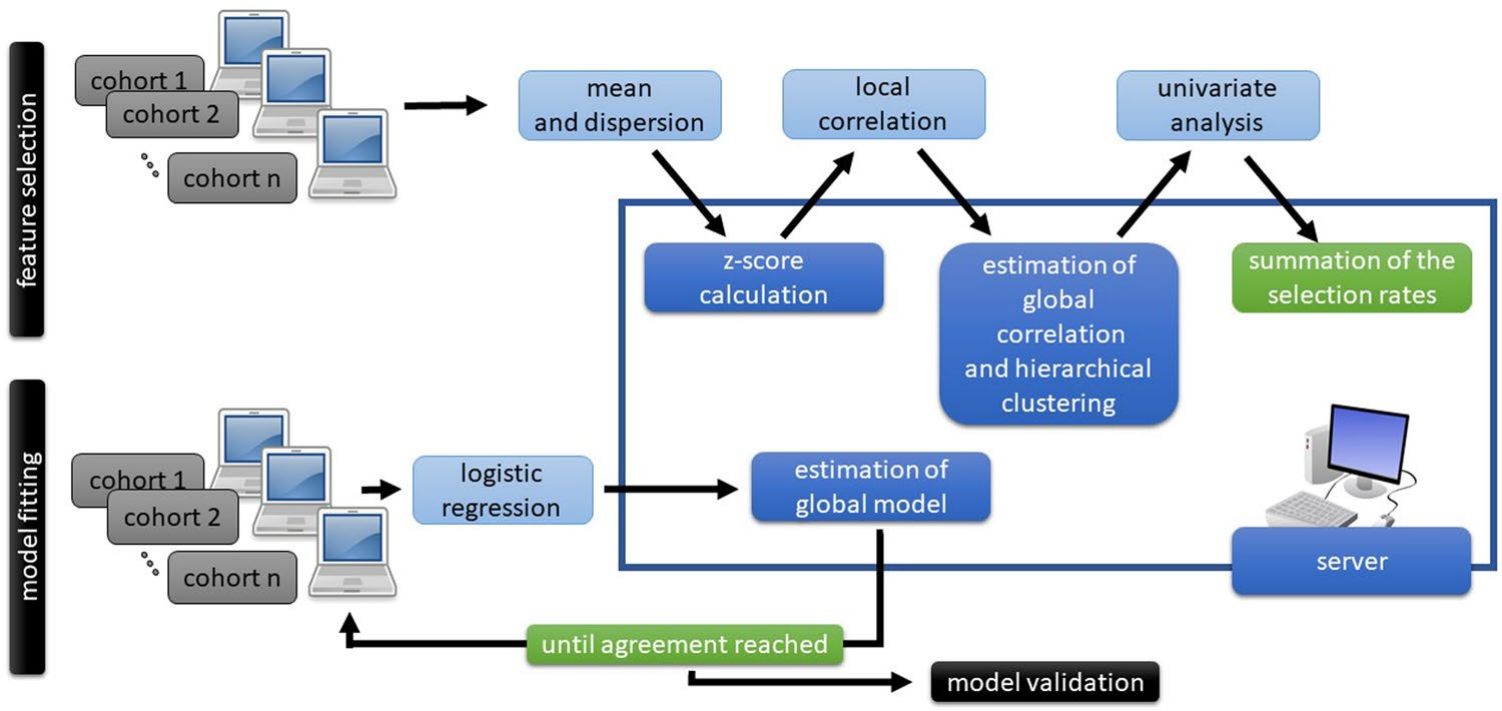
Scheme of the distributed model training. Model training was divided into two parts: feature selection and model fitting. In both parts local statistics were computed at the local repositories and sent to the central server. In the central server the global statistics were estimated and sent back to the local repositories. Finally, the model was tested in a validation cohort.

Next, inter-features correlations were assessed (Fig. 1). Features were scaled with the z-score. In distributed learning, the global mean and standard deviation per feature were obtained by sharing local statistics on mean, dispersion from mean and number of patients in the cohort. The global correlations were estimated as weighted average of fisher transformed local correlation coefficients. The average linkage hierarchical clustering (Python SciPy library v. 1.3.0) was performed on the set of inter-features correlation coefficients with a 0.6 cutoff, separately for the centralized and distributed learning.

Finally, to select a feature representative per cluster a univariate logistic regression was performed on the entire dataset (centralized learning) as well as the separate cohorts (distributed learning). In the centralized learning, per cluster, the feature with the highest area under the receiver operator characteristic curve (AUC) was chosen if the false discovery rate <0.05. In the distributed learning, per cohort and per cluster, the feature with the highest AUC was chosen to represent each cluster. In the central sever the cohort-specific sets were compared and weighted by the number of patients in the cohort. The final distributed feature selection comprised features with at least 80% selection rate, based on cohort sizes as weights.

### Classification

A multivariate logistic regression model was trained for both outcomes, HPV and 2 year overall survival (2yOS). In the centralized learning, the model was fitted with a GLM (generalized linear models) function in R (version 3.2.3). In the distributed learning, the grid binary logistic regression (GLORE) method was used to fit the coefficients^18^. It is based on the intermediate agglomeration of the Newton-Raphson solutions. It has been previously shown to estimate the coefficients well in the horizontally partitioned datasets^18^.

### Comparison of the models

Five models were created to predict HPV status and another five to predict 2yOS. For each of the models, four cohorts were used for training and one was left out for external validation (patients with unknown status were excluded from modeling of the respective outcome). The prognostic power of a model was evaluated in the validation cohort. Models were trained in a distributed and centralized manner for comparison.

The comparison was divided in three parts. First, the feature selection was evaluated. The overlap in class assignments in hierarchical clustering was computed. Features were divided into subgroups based on the centralized clustering and next, on the cluster by cluster basis, the largest distributed subcluster was reported. Sum of features in the distributed subclusters divided by total number of features was defined as cluster overlap. To quantify the impact of feature selection on the prognostic power of the model, the glm function was used to fit the model based on centralized and distributed feature selection. The area under receiver operating characteristics (AUC) from the following models were compared with a DeLong test (p-value < 0.05). Additionally, overlap between the selected features was reported. In the second step, model fitting was compared. The models based on distributed feature selection were created with glm and GLORE. The quality of fit (loglikelihood) was reported. The performance of models was evaluated with DeLong test. Finally, the full process (feature selection and classification) was compared. ROC curves were evaluated and model calibration in the validation cohort was checked. Calibration was estimated by fitting a logistic regression model in the validation cohort with one variable - predictions based on the model from the training cohort. The model was considered well-calibrated, if the obtained coefficient was not significantly different from 1. The calibration on a feature-basis was not analyzed. The patients were split into two groups (HPV+/−, and OS risk groups) based on the median prediction in the training cohort and group assignments between centralized and distributed models were compared $({\rm{classification}}\,{\rm{discrepancy}}\,=$ ${\rm{number}}\,{\rm{of}}\,{\rm{patients}}\,{\rm{assigned}}\,{\rm{to}}\,{\rm{different}}\,{\rm{classes}}/{\rm{total}}\,{\rm{number}}\,{\rm{of}}\,{\rm{patients}}\,{\rm{in}}\,{\rm{the}}\,{\rm{validation}}\,{\rm{cohrot}})$. Additionally, for the 2yOS model, the Kaplan-Meier curves were plotted, using a median split to divide patients into risk groups.

## Results

### Centralized vs distributed feature selection

Close to 20% of radiomic features were excluded in the data cleaning process due to missing values or highly skewed distribution (details presented in Supplementary Table S7 and S8), irrespective of modeling endpoint and centralized or distributed cleaning. The remaining features were independently clustered using centralized and distributed correlation coefficients. Here we present the respective values as a range, depending on the results from different training/validation cohorts. The centralized clustering resulted in a slightly higher number of clusters 97–103 vs 90–95 for HPV and 105–113 vs 94–98 for 2yOS. Depending on the studied cohorts combination 94–97% of the features were clustered in the same groups in the centralized and distributed clustering.

For tumor HPV status prediction, 26–30 and 12–28 features were selected in the centralized and distributed way, respectively. The overlap of selected features between the methods was around 50%. Less variability in the number of selected features was observed in the case of 2yOS endpoint, with 10–21 and 7–23 features in the centralized and distributed selection, respectively. However, the overlap was lower, on average 40%. Detailed comparison is presented in Supplement Fig. 1S and Tables 2.1S–3.5S.

**Figure 2:**
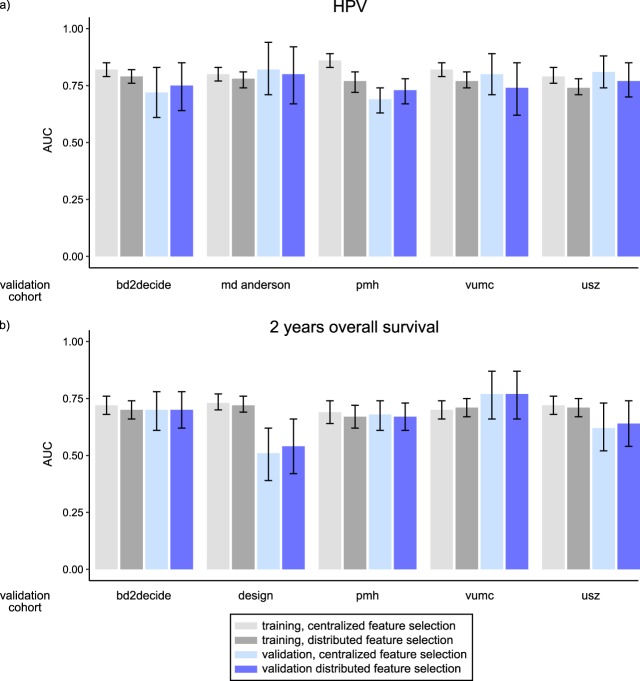
Comparison of feature selection methods based on the area under receiver operating characteristics (AUC). The bars present results from both centralized (light gray and light blue) and distributed (dark grey and dark blue) feature selection together with 95% confidence intervals. No statistically significant difference was observed between the selection methods (DeLong p-value> 0.05).

Figure 2 presents the summary of performance (AUC) of models trained on the feature subsets selected in the centralized and distributed workflows, for both HPV (a) and 2yOS (b). The model coefficients were trained with glm in both cases. No significant difference in AUC was observed (DeLong p-value> 0.05), indicating that a lower number of radiomic features in the distributed selection does not decrease model performance.

The 2yOS model validation failed in the DESIGN cohort. However, this is the only cohort with solely HPV negative patients. To further check the influence of HPV on our 2yOS models, we validated the 2yOS models in the oropharyngeal carcinoma cohorts for subgroups of HPV+ and HPV−. They showed good prognostic value in both subgroups, with AUC in a range of 0.61 to 1 (Supplementary Table 4S).

### Centralized vs distributed logistic regression

**Figure 3:**
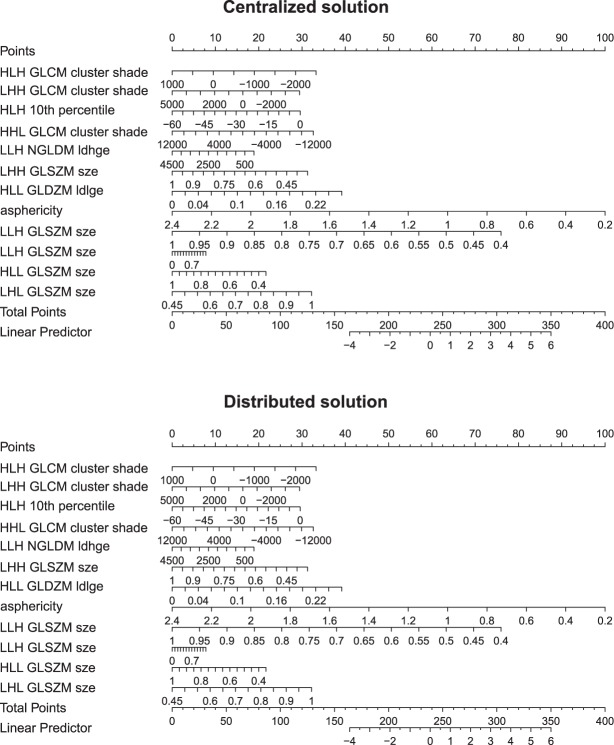
Comparison of nomograms for models obtained using centralized and distributed logistic regression. The coefficients of the models are identical. Example for the model prediction HPV, trained on the cohorts: bd2decide, md anderson, vumc, usz.

The logistic regression fits were compared based on the subset of features selected in the distributed manner. The glm and GLORE algorithms reached identical log-likelihood for all training cohorts combinations and both endpoints (Supplementary Tables 5S and 6S). The sum of absolute differences in the coefficients between the centralized and distributed solution was less than 10^−7^. Figure 3 presents an example of nomograms obtained using the centralized and distributed logistic regression for HPV prediction.

### Centralized vs distributed models

**Figure 4:**
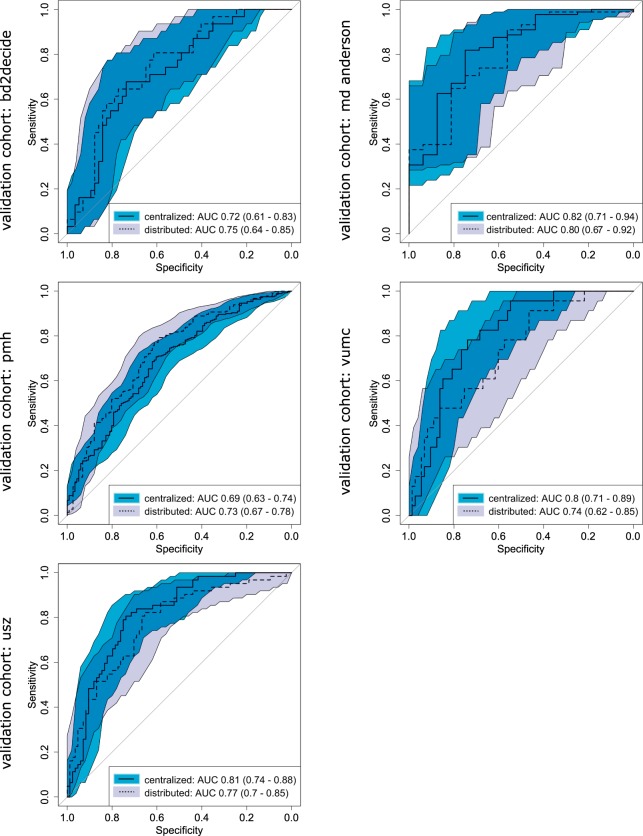
The receiver operating characteristics of radiomics-based models for HPV prediction. The AUCs are given with 95% confidence interval. No significant difference in ROC was observed between models trained in the centralized and distributed workflow.

**Figure 5:**
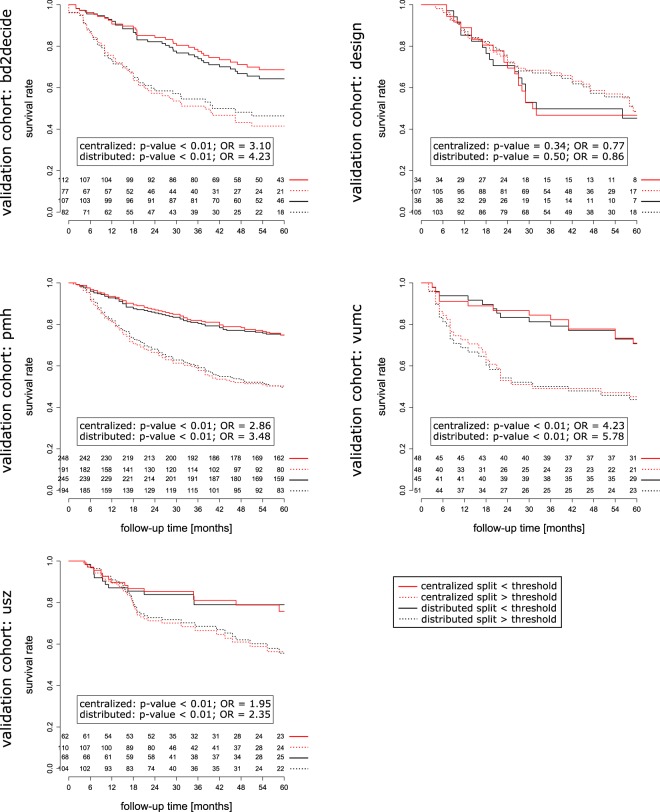
Comparison of Kaplan-Meier curves for the risk-group split based on the 2 years overall survival models trained centrally and distributed. Both models performed equally well on all validation cohorts. The G-rho test p-values and odds ratio (OR) are shown for comparison.

In the final comparison, results from both centralized and distributed workflows were evaluated in the validation cohorts. The HPV prediction models performed equally good in terms of discriminatory power in the centralized and distributed learning (Fig. 4, Table 5S). However, 18–28% classification discrepancy was observed between the centralized and distributed models, when median prediction in training dataset was used as threshold. Also, no significant difference in the discriminatory power was observed for models predicting 2yOS. Additionally, both centralized and distributed risk-group split thresholds were significant for all validation cohorts, except DESIGN cohort (Figs. 5 and 2S, Table 6S). The resulting Kaplan-Meier curves followed the same trend. Similarly, to the HPV models, classification discrepancy of 13–21% was observed between centralized and distributed model. In total, 12 out of 20 models (HPV and OS) would have required recalibration in the validation cohort (logistic regression coefficient significantly different from 1), however it was not dependent on the training workflow (Table 6S). Recalibration was not performed as part of this study and the results of split into risk groups for 2yOS model were based on the original predictions.

## Discussion

This study aimed at designing and testing of a distributed learning workflow using radiomics data. CT images from more than 1000 HNC patients were analyzed with HPV status and 2 year overall survival prediction as endpoints. Combination of hierarchical clustering and univariate logistic regression was used for feature selection, and multivariate logistic regression was used for final classification. The resulting models obtained with distributed learning were compared to the centrally trained models. Models for both endpoints showed comparable results in the centralized and distributed training, on the level of feature selection, model fitting as well as the full workflow comparison.

Other studies have investigated horizontal data partitioning and distributed feature selection mostly with a focus on higher computational efficiency^15,16,19^. Here we present a simple algorithm based on the assumption that the distribution of radiomic feature values is similar in all studied cohorts. Although this assumption may not always be correct due to different image acquisition protocols^7,20,21^, we observed a good agreement between centralized and distributed clustering. Of note, the selection of features using majority voting among the cohorts may decrease the risk of selecting cohort-specific or scanner-specific biomarkers. The overlap between the final feature selection (distributed vs centralized) was not high but this could be caused by strong inter-features correlations or redundancy of the selected features as no stepwise feature selection was included in the multivariate model training. No difference in model performance was observed depending on the feature selection manner.

Several previous studies have investigated distributed classification algorithms in the healthcare data, presenting satisfactory results in terms of model accuracy^12–14^. The GLORE algorithm used in this study provided excellent results with a fast convergence (less than 10 iterations). In the comparison of the entire workflows, the difference in the AUCs between the centralized and distributed models was smaller than the AUCs dispersion resulting from different combination of training data. In the HPV models the largest difference between centralized and distributed learning was 0.07, whereas the observed range of AUCs depending on the training data was 0.69–0.82. We observed 18–28% classification discrepancy between our centralized and distributed models. The median threshold was used to classify patients, other splits should be evaluated in the future.

CT radiomics has previously been evaluated for prediction of overall survival and HPV status^22–25^. The performance of the distributed HPV models (AUC 0.73–0.80) is comparable with previously published results (AUC 0.70–0.80). In this study, the HPV prediction was performed for all patient with available data and was not limited to the oropharyngeal cancer, which would be more relevant in the clinical practice. In the context of overall survival, Parmar *et al*. reported an AUC of 0.61–0.67 depending on the used classifier^25^. This study was able to achieve similar model performance in distributed learning even using a fixed classification method (AUC 0.64–0.77). One exception was observed for the model trained on a mixed cohort of head and neck cancer patients and validated on the HPV- cohort (DESIGN cohort), for both centralized and distributed learning. Recent literature provides extensive evidence on superior survival rates of HPV positive oropharyngeal cancer patients^26–28^. We have shown in other combinations of training data that our overall survival models were prognostic in both HPV+ and HPV− oropharyngeal cancer (Table 4S). This would indicate that the models were not driven by HPV status and radiomics can be used as biomarker for both disease subtypes. However to fully exploit potential of radiomics, matched data should be used for model training, i.e. only HPV− patients. The access to large databases does not replace careful data curation.

Currently, implementation of distributed learning into healthcare is still at an early stage. There is a need to build trust between hospitals, IT departments and ethical committees to allow for integration of distributed learning network into the clinical picture archiving and communication systems and reporting systems. From the technological perspective, integration of distributed learning is feasible, two commercial solution supporting distributed learning infrastructure are available DistriM from Oncoradiomics and Varian Learning Portal from Varian as well as open source solutions^29^. In the DistriM solution, which is compatible with the algorithms developed for this study, data are secured by storing them on computer systems within the firewalls of the hospital. Only model coefficients are transmitted, from which individual patient characteristics cannot be derived.

Our study is the first attempt to combine radiomics data and distributed learning. For the comparison purpose, all data were collected at the same location and data quality assurance as well as radiomic features were extracted by one person. This experiment was a proof of concept that radiomics-based models can be trained in the distributed fashion. However, all the algorithms developed in this work are compatible with DistriM framework. Due to the experiment design we were not able to evaluate important aspects of real-life distributed learning scenario, such as speed, security and network issues. Moreover, in the multicenter setting, simple data quality checks should be implemented, for example reporting of maximum and minimum intensity in the region of interest to avoid major contour shifts. The standardization of radiomic features extraction is currently ongoing. If future studies will decide to use mixed software implementations (separate implementation in each of the learning sites), an ontology for radiomics has to be defined and the implementations have to be benchmarked, for example in the Imaging Biomarker Standardization Initiative^30–33^. Additionally, multicenter data analysis requires efforts in establishing post-processing steps for data standardization, as for example contrast-enhancement normalization^34^ or robustness studies on contouring variability^7,17,20,33,35^. Our models showed good discrimination, but in 12/20 cases would require recalibration. This is a challenge in the transfer of the trained models into a new institution or scanner. For the quality assurance, such model should be first validated on sample of data in the new institution/scanner (if needed recalibrated) and only then used in prospective setting. Despite feature preselection, the final models consisted of 7–28 features, which might have resulted in inclusion of redundant features into the multivariate model. The next step in the development of distributed radiomics workflow could be integration of stepwise regression. Additionally, in the future easy access to radiomics data via distributed learning will allow for regular updates (e.g. yearly) of the studied signatures to further prove that they are not study time dependent or whether they are applicable for new treatment modalities^36^. Finally, we would like to apply distributed learning to various clinically relevant outcomes, such as treatment failure, early death and hypoxia status^37–39^ and compare distributed learning radiomics to results from distributed deep learning^40^.

In conclusion, this study describes the first workflow for radiomics analysis in a distributed setting. Centralized and distributed learning results for prediction of HPV status and 2 year overall survival in HNSCC patients treated with radical chemoradiotherapy or radiotherapy were similar. This methodology will allow for easier access to radiomics data from large cohorts and thus development of more robust and reliable models. This approach will also facilitate regular updates of radiomics signatures when new treatment or imaging modalities are implemented.

## Supplementary information

Supplementary material.
