# Supplementary material for: Privacy-preserving distributed learning of radiomics to predict overall survival and HPV status in head and neck cancer
Source: Sci Rep. 2020 Mar 11;10:4542. doi: 10.1038/s41598-020-61297-4 (PMC7066122; doi:10.1038/s41598-020-61297-4)
Supplement: Supplementary file 1 — Supplementary material. [file 41598_2020_61297_MOESM1_ESM.pdf]

Supplementary material: Privacy-preserving distributed learning of radiomics to predict overall survival and HPV status in head and neck cancer

Marta Bogowicz, Arthur Jochems, Timo M. Deist, Stephanie Tanadini-Lang, Shao Hui Huang, Biu Chan, John N Waldron, Scott Bratman, Brian O'Sullivan, Oliver Riesterer, Gabriela Studer, Jan Unkelbach, Samir Barakat, Ruud H Brakenhoff, Irene Nauta, Silvia E Gazzani, Giuseppina Calareso, Kathrin Scheckenbach, Frank Hoebers, Frederik WR Wesseling, Simon Keek, Sebastian Sanduleanu, Ralph TH Leijenaar, Marije R. Vergeer, Rene C Leemans, Chris HJ Terhaard, Michiel WM van den Brekel, Olga Hamming-Vrieze, Martijn A van der Heijden, Hesham M Elhalawani, Clifton D Fuller, Matthias Guckenberger, Philippe Lambin

Ethics approval

- BD2DECIDE - data analysis was approved by and was carried out in accordance to its regulation. All patients signed informed consent.
- DESIGN - the collection of patient data and images was according to the guidelines of the Dutch Medical Scientific Societies (www.federa.org), and provided pseudo-anonimized under a data agreement.
- MD Anderson - patients were retrieved from an internal University of Texas MD Anderson Cancer Center database after getting approved by the University of Texas MD Anderson Cancer Center Institutional review board (IRB). All methods for this study were performed in accordance with the University of Texas MD Anderson Cancer Center IRB guidelines and regulations. Being a Health Insurance Portability and Accountability Act of 1996 (HIPAA) - compliant retrospective study waived the prerequisite for informed consent.
- PMH – data analysis was approved by University Health Network (Princess Margaret Cancer Centre). It is a retrospective chart review and individual patient's consent is waived.
- VUmc – the collection of patient data and images was according to the guidelines of the Dutch Medical Scientific Societies (www.federa.org), and provided pseudo-anonimized under a data agreement.
- USZ – data analysis was approved by the Swissethics and was carried out in accordance with Swissethics guidelines and regulations. Patients in the retrospective (n = 118) and prospective (n = 58) manner. Patients in retrospective cohort gave informed general consent and informed specific consent was obtained from the prospective cohort.

Results

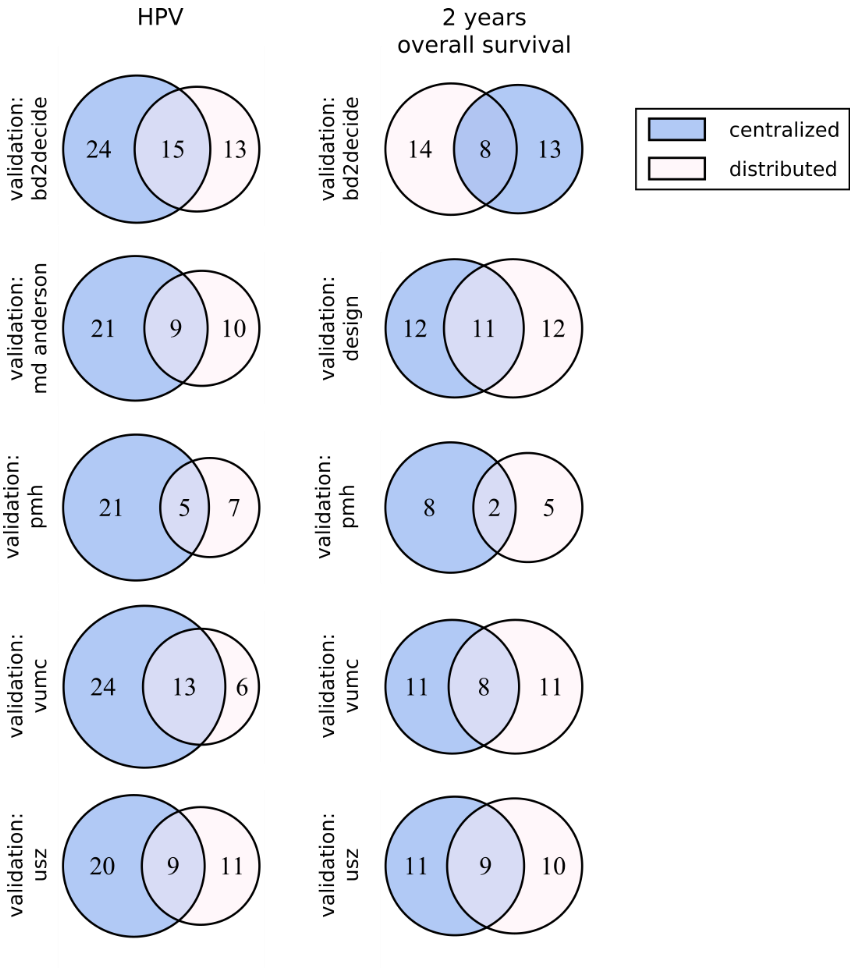

Figure 1S. Venn diagrams for the comparison of the features selected in the centralized and distributed workflow.

	center	BD2DECIDE	DESIGN	MD Anderson	PMH	VUmc	USZ
	number of patients	206	141	110	441	100	176
**scanner**	GE Medical Systems, Light Speed	28	17	104	65	74	---
	GE Medical Systems, Discovery	44	48	1	242	---	13
	GE Medical Systems, Bright Speed	1	---	---	---	---	---
	Siemens Biograph	53	24	---	---	---	---
	Siemens SOMATOM	42	---	---	---	9	110
	Siemens, Volume Zoom	10	---	---	---	---	47
	Siemens, Sensation	15	---	1	---	---	---
	Toshiba, Aquilion	---	---	1	132	---	---
	Toshiba, Asteion	1	---	---	---	---	---
	Philips, Brilliance	5	45	2	2	---	---
	Philips, Gemini	5	4	1	---	---	---
	Philips, Aura	1	1	---	---	---	---
	Philips, mx8000	1	---	---	---	---	---
	unknown	---	2	---	---	17	4
**adaptive mAs**	yes	n = 137<br>262 mAs<br>(45-508)	n = 99<br>372 mAs<br>(60-480)	n = 102<br>277 mAs<br>(220-582)	n = 122<br>396 mAs<br>(358-455)	n = 71<br>278 mAs<br>(223-365)	n = 23<br>229 mAs<br>(104-275)
	no	n = 69<br>167 mAs<br>(100-445)	n = 42<br>163 mAs<br>(100-450)	n = 8<br>220 mAs<br>(155-499)	n = 319<br>300 mAs<br>(273-500)	n = 29<br>200 mAs<br>(159-325)	n = 153<br>214 mAs<br>(60-450)
**kV**		80;120;130; 140	120;140	120; 140	120	120; 140	120; 140
**slice thickness [mm]**		0.7-4.0	1.0-2.5	1.0–3.0	2.0	1.5–3.0	1.5-3.3
**in-plane resolution [mm]**		0.98<br>(0.32-1.34)	0.83<br>(0.47-1.27)	0.48<br>(0.43–0.55)	0.98<br>(0.81-1.17)	0.86<br>(0.67–0.98)	0.98<br>(0.84-1.95)

Table 1S. Detials of the CT imaging protocols.

training: bd2decide, md anderson, pmh, vumc, usz	
**centralized feature selection**	**distributed feature selection**
LLL GLSZM small size emphasis	LLL GLSZM small size emphasis
LLH GLCM energy	LLH GLCM energy
histogram skewness	histogram skewness
HHH GLSZM small size emphasis	HHH GLSZM small size emphasis
HLH GLSZM small size emphasis	HLH GLSZM small size emphasis
HLL GLSZM small size emphasis	HLL GLSZM small size emphasis
LHH GLSZM small size emphasis	LHH GLSZM small size emphasis
LLH GLSZM small size emphasis	LLH GLSZM small size emphasis
shape flatness	shape flatness
HHL histogram energy	HHL histogram energy
shape sphericity	shape sphericity
LLH histogram entropy	LLH histogram entropy
HHH GLDZM large distance low gray-level emphasis	HHH GLDZM large distance low gray-level emphasis
LLL histogram coefficient of variation	LLL histogram coefficient of variation
LLL GLCM difference entropy	LLL GLCM cluster shade
LLL NGLDM small dependence high gray-level emphasis	HHL histogram percentile 10th
GLCM entropy	HLL GLSZM small size high gray-level emphasis
NGLDM large dependence high gray-level emphasis	HLL histogram percentile 10th
HLL histogram entropy	LLH GLCM information measures of correlation 2
LHL NGLDM small dependence high gray-level emphasis	HLH histogram percentile 10th
HLL NGLDM small dependence high gray-level emphasis	LLL GLSZM large size low gray-level emphasis
LLH histogram percentile 10th	HHL GLSZM small size emphasis
HLL GLCM dissimilarity	LHL histogram interquartile range
LHH GLCM dissimilarity	HHH histogram skewness
HLH NGLDM dependence count non-uniformity	LLH GLCM cluster shade
LLL GLCM inverse difference	LLH GLCM joint average
LLL GLCM cluster prominence	HHH GLCM homogeneity normalized
HLH GLDZM small distance low gray-level emphasis	GLSZM small size emphasis
LLL NGTDM complexity	
LLL GLDZM small distance low gray-level emphasis	
HLH histogram percentile 90th	
HLL NGTDM complexity	
LHH NGTDM complexity	
HLH NGTDM complexity	
HHH NGTDM complexity	
LHL NGTDM complexity	
LLH GLCM sum of average	
LLH NGTDM complexity	

Table 2.1S. Comparison of the features selected in the centralized and distributed workflow for HPV prediction in the training cohort of: md anderson, pmh, vumc, usz (all ~ bd2decide)

training: bd2decide, pmh, vumc, usz	
**centralized feature selection**	**distributed feature selection**
LLL GLSZM small size emphasis	LLL GLSZM small size emphasis
histogram skewness	histogram skewness
HLL GLSZM small size emphasis	HLL GLSZM small size emphasis
LLH GLSZM small size emphasis	LLH GLSZM small size emphasis
LLL NGLDM small dependence high gray-level emphasis	LLL NGLDM small dependence high gray-level emphasis
shape sphericity	shape sphericity
HHH GLDZM large distance low gray-level emphasis	HHH GLDZM large distance low gray-level emphasis
LLL histogram coefficient of variation	LLL histogram coefficient of variation
HHL histogram percentile 10th	HLL NGLDM small dependence high gray-level emphasis
LLL GLCM variance	LLH GLCM joint average
HLL histogram uniformity	LLL GLSZM large size low gray-level emphasis
LLH GLCM joint maximum	HHL GLSZM small size emphasis
LLL NGTDM complexity	LHH GLSZM small size emphasis
HLH NGLDM dependence count non-uniformity	shape flatness
LHL NGTDM complexity	LLH GLCM cluster shade
HLH histogram uniformity	HHH histogram skewness
NGTDM contrast	HLH GLRLM long run low gray-level emphasis
HHH histogram percentile 90th	GLSZM small size emphasis
HHL NGTDM complexity	LLL GLCM cluster shade
LHL histogram interquartile range	
HLL histogram interquartile range	
HLH histogram mean absolute deviation	
LHH histogram mean absolute deviation	
LLH histogram mean absolute deviation	
HLH GLCM entropy	
GLDZM large distance high gray-level emphasis	
LHL NGLDM small dependence high gray-level emphasis	
HLL NGTDM complexity	
LLH histogram percentile 10th	

Table 2.2S. Comparison of the features selected in the centralized and distributed workflow for HPV prediction in the training cohort of: bd2decide, pmh, vumc, usz (all ~ md anderson)

training: bd2decide, md anderson, vumc, usz	
**centralized feature selection**	**distributed feature selection**
LLH NGLDM small dependence high gray-level emphasis	LLH NGLDM small dependence high gray-level emphasis
LLH GLSZM small size emphasis	LLH GLSZM small size emphasis
HLL GLSZM small size emphasis	HLL GLSZM small size emphasis
LHH GLSZM small size emphasis	LHH GLSZM small size emphasis
LLL GLSZM small size emphasis	shape sphericity
LLL GLCM cluster prominence	HLH histogram percentile 10th
GLDZM gray-level non-uniformity	LLL GLSZM large size low gray-level emphasis
histogram skewness	LHL GLSZM small size emphasis
HLH GLCM energy	HHL GLCM cluster shade
LHH NGTDM complexity	HLH GLCM cluster shade
histogram coefficient of variation	LHH GLCM cluster shade
LLH NGTDM complexity	HLL GLDZM large distance low gray-level emphasis
HLL NGTDM complexity	
LLL histogram uniformity	
GLCM inverse difference	
HLH NGTDM complexity	
LLL histogram percentile 90th	
LLL NGLDM small dependence high gray-level emphasis	
shape flatness	
HLL GLCM homogeneity	
LLH histogram entropy	
LHH histogram entropy	
HLL histogram entropy	
LLH histogram percentile 10th	
LLL histogram skewness	

Table 2.3S. Comparison of the features selected in the centralized and distributed workflow for HPV prediction in the training cohort of: bd2decide, md anderson, vumc, usz (all ~ pmh)

training: bd2decide, md anderson, pmh, usz	
**centralized feature selection**	**distributed feature selection**
LLL GLSZM small size emphasis	LLL GLSZM small size emphasis
HHL histogram percentile 10th	HHL histogram percentile 10th
histogram skewness	histogram skewness
LLH GLCM information measures of correlation 2	LLH GLCM information measures of correlation 2
HLL GLSZM small size emphasis	HLL GLSZM small size emphasis
LHH GLSZM small size emphasis	LHH GLSZM small size emphasis
LLH GLSZM small size emphasis	LLH GLSZM small size emphasis
LHL histogram interquartile range	LHL histogram interquartile range
shape flatness	shape flatness
LLH GLCM joint average	LLH GLCM joint average
shape sphericity	shape sphericity
GLSZM small size emphasis	GLSZM small size emphasis
LHH NGTDM complexity	HHH GLDZM large distance low gray-level emphasis
HHH GLSZM small size emphasis	HLL GLSZM small size high gray-level emphasis
HLH GLSZM small size emphasis	LLL GLSZM large size low gray-level emphasis
HLL histogram interquartile range	HHL GLSZM small size emphasis
LLL NGLDM small dependence high gray-level emphasis	LLH GLCM cluster shade
HHL NGLDM small dependence high gray-level emphasis	LLL GLCM cluster shade
LLH GLCM contrast	HLH GLCM inverse variance
LHL NGLDM small dependence high gray-level emphasis	
HLL NGLDM small dependence high gray-level emphasis	
HLH histogram percentile 10th	
LLH histogram percentile 10th	
LHH histogram percentile 10th	
HLH GLRLM gray-level non-uniformity	
HLL GLRLM gray-level non-uniformity	
HLH histogram mean absolute deviation	
LHH histogram mean absolute deviation	
HLH NGLDM dependence count non-uniformity	
LLL histogram coefficient of variation	
LLL GLCM inverse difference	
LLL GLCM cluster prominence	
LHL NGTDM complexity	
HLL NGTDM complexity	
HHH histogram percentile 90th	
HHL NGTDM complexity	

Table 2.4S. Comparison of the features selected in the centralized and distributed workflow for HPV prediction in the training cohort of: bd2decide, md anderson, pmh, usz (all ~ vumc)

training: bd2decide, md anderson, pmh, vumc	
**centralized feature selection**	**distributed feature selection**
LLL GLSZM small size emphasis	LLL GLSZM small size emphasis
LLH GLCM information measures of correlation 2	LLH GLCM information measures of correlation 2
LLH NGTDM complexity	LLH NGTDM complexity
HLH GLSZM small size emphasis	HLH GLSZM small size emphasis
HLL GLSZM small size emphasis	HLL GLSZM small size emphasis
LLH GLSZM small size emphasis	LLH GLSZM small size emphasis
HHL histogram energy	HHL histogram energy
shape sphericity	shape sphericity
LLL NGTDM complexity	HHH GLDZM large distance low gray-level emphasis
LHH NGTDM complexity	HLH histogram percentile 10th
histogram skewness	LLL GLSZM large size low gray-level emphasis
LHL GLDZM small distance low gray-level emphasis	HHH GLSZM small size emphasis
HLH NGLDM dependence count non-uniformity	HHL GLSZM small size emphasis
histogram coefficient of variation	LHH GLSZM small size emphasis
LHL NGTDM complexity	LLH GLRLM short run low gray-level emphasis
HLL NGTDM complexity	shape flatness
LHH histogram percentile 10th	LLH GLCM cluster shade
HLH NGTDM complexity	LLH GLCM joint average
HLL histogram interquartile range	GLSZM small size emphasis
NGTDM complexity	LLL GLCM cluster shade
LLL NGLDM small dependence high gray-level emphasis	
LLL histogram uniformity	
LLH histogram entropy	
HLL GLRLM gray-level non-uniformity	
LHL NGLDM small dependence high gray-level emphasis	
LLL GLCM inverse difference	
HLL NGLDM small dependence high gray-level emphasis	
histogram entropy	

Table 2.5S. Comparison of the features selected in the centralized and distributed workflow for HPV prediction in the training cohort of: bd2decide, md anderson, pmh, vumc (all ~ usz)

training: design, pmh, vumc, usz	
**centralized feature selection**	**distributed feature selection**
HLH histogram energy	HLH histogram energy
HHL histogram energy	HHL histogram energy
HHH histogram energy	HHH histogram energy
LLH histogram energy	LLH histogram energy
LHH histogram energy	LHH histogram energy
HLL histogram energy	HLL histogram energy
LLH GLCM joint average	LLH GLCM joint average
shape sphericity	LLH GLCM energy
LHL GLDZM large distance high gray-level emphasis	LHL GLCM inverse difference normalized
HLL GLDZM zone distance variance	HLL histogram percentile 10th
HLL GLSZM zone size entropy	LHH histogram percentile 10th
LLL NGTDM complexity	HHH GLSZM small size emphasis
LHH GLDZM large distance high gray-level emphasis	HLH GLSZM small size emphasis
LLL GLDZM zone distance variance	HLL GLSZM small size emphasis
LLL GLDZM large distance high gray-level emphasis	HHL NGLDM low gray-level count emphasis
HLH GLCM joint average	LLH GLSZM small size emphasis
HLL NGLDM dependence count non-uniformity	GLCM entropy
LHL histogram energy	HHH histogram skewness
GLCM information measures of correlation 1	LHH GLCM inverse difference normalized
HLL histogram entropy	GLSZM small size emphasis
LLL histogram skewness	LHL GLCM maximal correlation coefficient

Table 3.1S. Comparison of the features selected in the centralized and distributed workflow for the 2 years overall survival prediction in the training cohort of: design, pmh, vumc, usz (all ~ b2decide)

training: bd2decide, pmh, vumc, usz	
**centralized feature selection**	**distributed feature selection**
LHL GLDZM large distance high gray-level emphasis	LHL GLDZM large distance high gray-level emphasis
HHL histogram range	HHL histogram range
LLL NGTDM complexity	LLL NGTDM complexity
LLL histogram range	LLL histogram range
HHH histogram energy	HHH histogram energy
LHL histogram energy	LHL histogram energy
LLH GLSZM small size emphasis	LLH GLSZM small size emphasis
HLH GLCM joint average	HLH GLCM joint average
LLH GLCM joint average	LLH GLCM joint average
shape sphericity	shape sphericity
HLL NGLDM dependence count non-uniformity	LLL GLSZM small size emphasis
LLH GLCM energy	LHL GLCM energy
HLL GLDZM zone distance variance	histogram skewness
HLL GLSZM zone size entropy	LHL GLDZM small distance low gray-level emphasis
HLH histogram energy	HHH GLCM autocorrelation
LLH histogram energy	HLH GLSZM small size emphasis
LHH GLDZM large distance high gray-level emphasis	HLL GLSZM small size emphasis
LHH histogram energy	LHH GLSZM small size emphasis
LLL GLDZM large distance high gray-level emphasis	LHH histogram percentile 90th
GLCM entropy	LLH GLCM cluster shade
LLH histogram entropy	HLH GLRLM long run low gray-level emphasis
HLL histogram entropy	GLSZM small size emphasis

Table 3.2S. Comparison of the features selected in the centralized and distributed workflow for the 2 years overall survival prediction in the training cohort of: bd2decide, pmh, vumc, usz (all ~ design)

training: bd2decide, design, vumc, usz	
**centralized feature selection**	**distributed feature selection**
LLL histogram range	LLL histogram range
HLH GLDZM distance zone non-uniformity	shape sphericity
LLH GLSZM zone size entropy	HLH GLSZM small size emphasis
HLH histogram energy	HHL GLSZM size zone non-uniformity
HHH histogram energy	LLH GLCM joint average
LLH histogram energy	GLSZM small size emphasis
LHH histogram energy	HLL NGLDM small dependence high gray-level emphasis
LLL GLDZM large distance high gray-level emphasis	
HLL histogram energy	

Table 3.3S. Comparison of the features selected in the centralized and distributed workflow for the 2 years overall survival prediction in the training cohort of: bd2decide, design, vumc, usz (all ~ pmh)

training: bd2decide, design, pmh, usz	
**centralized feature selection**	**distributed feature selection**
LHL GLDZM large distance high gray-level emphasis	LHL GLDZM large distance high gray-level emphasis
HLH histogram energy	HLH histogram energy
LLL histogram range	LLL histogram range
HHH histogram energy	HHH histogram energy
HLL histogram energy	HLL histogram energy
HLH GLCM joint average	HLH GLCM joint average
LLH GLCM joint average	LLH GLCM joint average
HLL GLDZM zone distance variance	LLL GLSZM small size emphasis
LLL NGTDM complexity	HHH GLCM autocorrelation
HLH NGLDM dependence count non-uniformity	histogram skewness
LLH histogram energy	HLH GLSZM small size emphasis
LHH GLDZM large distance high gray-level emphasis	HLL GLSZM small size emphasis
LHH histogram energy	LHH GLSZM small size emphasis
LHL histogram energy	LLH GLSZM small size emphasis
GLCM entropy	LLH GLCM cluster shade
LLL GLDZM large distance high gray-level emphasis	HLH GLRLM long run low gray-level emphasis
HLH histogram entropy	GLSZM small size emphasis
HLH GLSZM zone size entropy	LHH histogram percentile 90th

Table 3.4S. Comparison of the features selected in the centralized and distributed workflow for the 2 years overall survival prediction in the training cohort of: bd2decide, design, pmh, usz (all ~ vumc)

training: bd2decide, design, pmh, vumc	
**centralized feature selection**	**distributed feature selection**
LHL GLDZM large distance high gray-level emphasis	LHL GLDZM large distance high gray-level emphasis
HLH histogram energy	HLH histogram energy
HHH histogram energy	HHH histogram energy
LHL histogram energy	LHL histogram energy
HLL histogram energy	HLL histogram energy
HLH GLCM joint average	HLH GLCM joint average
LLH GLCM joint average	LLH GLCM joint average
shape sphericity	shape sphericity
HLL GLDZM zone distance variance	LLL GLSZM small size emphasis
HHL histogram range	HHH GLCM autocorrelation
LLH GLSZM zone size entropy	histogram skewness
HLH NGLDM dependence count non-uniformity	LLL NGTDM complexity
LLL histogram range	LHH histogram percentile 10th
LLH histogram energy	HLH GLSZM small size emphasis
LHH GLDZM large distance high gray-level emphasis	HLL GLSZM small size emphasis
LHH histogram energy	LLH GLCM cluster shade
LLL GLDZM large distance high gray-level emphasis	GLSZM small size emphasis
LLH histogram entropy	LHH NGLDM small dependence low gray-level emphasis
HHH GLDZM large distance low gray-level emphasis	

Table 3.5S. Comparison of the features selected in the centralized and distributed workflow for the 2 years overall survival prediction in the training cohort of: bd2decide, design, pmh, vumc (all ~ usz)

validation						
	AUC	95% CI		AUC	95% CI	
**pmh**	0.61	0.51	0.72	0.63	0.54	0.72
**vumc**	0.67	0.55	0.80	1*	---	---

Table 4S. Validation of the 2 years overall survival models on the HPV+ and HPV- subgroups of oropharyngeal cancer. * in this subgroup there was only one patient who died.

validation center		bd2decide		md anderson		pmh		vumc		usz	
		centralized	distributed	centralized	distributed	centralized	distributed	centralized	distributed	centralized	distributed
**training**	AUC	0.82	0.79	0.80	0.78	0.86	0.77	0.82	0.77	0.79	0.74
	95% CI	0.79	0.76	0.77	0.74	0.83	0.72	0.79	0.74	0.76	0.71
		0.85	0.82	0.83	0.81	0.89	0.81	0.85	0.81	0.83	0.78
	loglikelihood	-398	-398	-404	-404	-249	-249	-396	-396	-390	-390
**validation**	AUC	0.72	0.75	0.82	0.80	0.69	0.73	0.80	0.74	0.81	0.77
	95% CI	0.61	0.64	0.71	0.67	0.63	0.67	0.71	0.62	0.74	0.70
		0.83	0.85	0.94	0.92	0.74	0.78	0.89	0.85	0.88	0.85
	DeLong (p-value)	0.32		0.51		0.12		0.08		0.34	
	calibration coefficient	0.42	0.25	0.36	0.73	0.31	0.32	0.19	-0.12	1.02	1.11
	calibration Hosmer-Lemeshow (p-value)	0.09	0.09	0.00	0.00	0.00	0.00	0.43	0.43	0.00	0.00
	classification discrepancy	0.18		0.22		0.28		0.21		0.20	

Table 5S. Details on HPV prediction models.

validation center		bd2decide		design		pmh		vumc		usz	
		centralized	distributed	centralized	distributed	centralized	distributed	centralized	distributed	centralized	distributed
**training**	AUC	0.72	0.70	0.73	0.72	0.69	0.67	0.70	0.71	0.72	0.71
	95% CI	0.68	0.66	0.70	0.69	0.64	0.62	0.66	0.67	0.68	0.67
		0.76	0.74	0.77	0.76	0.74	0.72	0.74	0.75	0.76	0.75
	loglikelihood	-423	-423	-443	-443	-324	-324	-467	-467	-437	-437
**validation**	AUC	0.70	0.70	0.51	0.54	0.68	0.67	0.77	0.77	0.62	0.64
	95% CI	0.61	0.62	0.39	0.42	0.61	0.61	0.66	0.66	0.52	0.54
		0.78	0.78	0.62	0.66	0.74	0.73	0.87	0.87	0.73	0.74
	DeLong (p-value)	0.98		0.39		0.88		0.95		0.68	
	calibration coefficient	0.76	0.76	0.80	0.94	1.06	1.12	0.79	0.75	0.97	0.94
	calibration Hosmer-Lemeshow (p-value)	0.14	0.11	0.00	0.01	0.03	0.13	0.08	0.03	0.11	0.16
	classification discrepancy	0.14		0.00		0.03		0.08		0.11	
	risk group split G-rho (p-value)	<0.01	<0.01	0.34	0.50	<0.01	<0.01	<0.01	<0.01	<0.01	<0.01

Table 6S. Details on the 2 years overall survival prediction models

validation cohort	number of excluded features						
	**bd2decide**	**design**	**pmh**	**vumc**	**zurich**	**distributed**	**centralized**
**zurich**	144	114	157	0		218	207
**vumc**	144	114	157		85	230	204
**pmh**	144	114		0	85	194	161
**design**	144		157	0	85	207	199
**bd2decide**	114		157	0	85	197	169

Table S7. Summary of the number of excluded features in the data cleaning process for overall survival modelling. The number of features is specified by cohort (columns), additionally the total number of excluded features is reported for the distributed and centralized data cleaning.

validation cohort	number of excluded features						
	**bd2decide**	**md anderson**	**pmh**	**vumc**	**zurich**	**distributed**	**centralized**
**zurich**	67	60	137	0		159	192
**vumc**	67	60	137		79	168	197
**pmh**	67	60		0	79	112	148
**md anderson**	67		137	0	79	157	193
**bd2decide**		60	137	0	79	162	165

Table S8. Summary of the number of excluded features in the data cleaning process for HPV modelling. The number of features is specified by cohort (columns), additionally the total number of excluded features is reported for the distributed and centralized data cleaning.

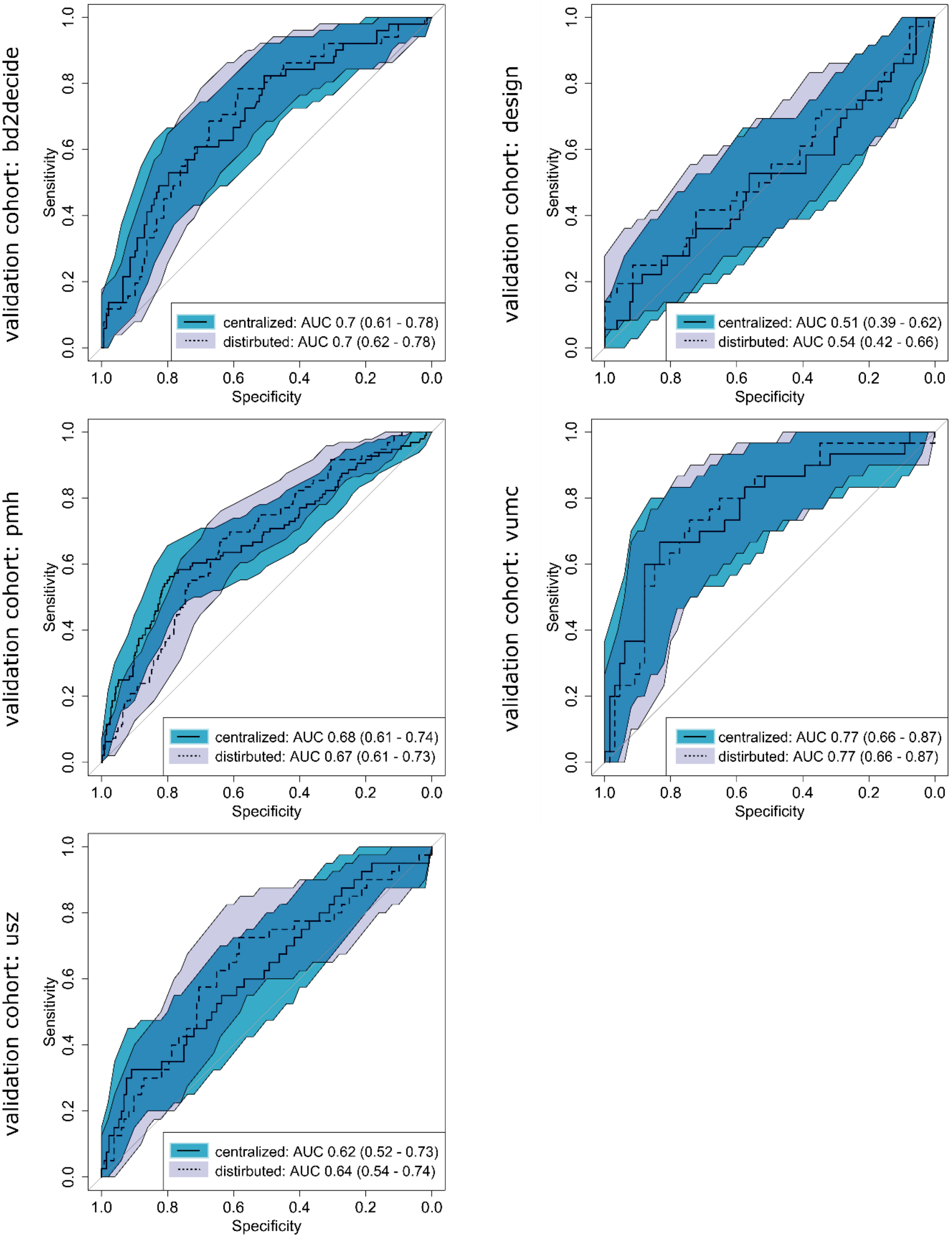

Figure 2S. The receiver operating characteristics of radiomics-based models for 2 years overall survival prediction. The AUCs are given with the 95% confidence interval. No significant difference was observed between models trained in the centralized and distributed workflow.
